# Development and comparison of evaluation metrics for batch correction reveals performance differences

**DOI:** 10.1093/bioadv/vbag142

**Published:** 2026-05-21

**Authors:** Aleksi Laiho, Marjaana Laitinen, Liisa Holm, Petri Törönen

**Affiliations:** Faculty of Biological and Environmental Sciences, University of Helsinki, Viikinkaari 1, P.O. Box 65, 00014 University of Helsinki, Finland; Faculty of Medicine, University of Helsinki, Haartmaninkatu 8, P.O. Box 63, 00014 University of Helsinki, Finland; Faculty of Biological and Environmental Sciences, University of Helsinki, Viikinkaari 1, P.O. Box 65, 00014 University of Helsinki, Finland; Faculty of Biological and Environmental Sciences, University of Helsinki, Viikinkaari 1, P.O. Box 65, 00014 University of Helsinki, Finland

## Abstract

**Motivation:**

Batch effects are a common challenge in the analysis of biological datasets, particularly RNA-seq data. Although numerous methods exist to correct batch effects, their comparative evaluation has received limited attention. Several metrics have been proposed to assess the effectiveness of batch correction, but it is unclear how consistently these metrics reflect performance. Here, we systematically investigate differences in the behavior and sensitivity of commonly used evaluation metrics for batch effect removal.

**Results:**

We compiled a set of established evaluation metrics and introduced several new metrics. These were systematically compared across multiple datasets generated using our Artificial Dilution Series approach: each dataset contained controlled levels of noise simulating batch effects, enabling quantitative assessment of each metric’s ability to discriminate between noise levels. We observed consistent differences among metrics, with those based on the F-statistic and the Davies–Bouldin index showing the strongest discriminative performance.

**Availability and implementation:**

The data and codes used in this article are available in https://github.com/Aleksi95/BatchMetrics.

## 1 Introduction

RNA sequencing analysis (RNA-Seq) is an increasingly important and widely used tool in the field of bioscience (Stark *et al.* 2019). However, reliable analysis of RNA-Seq data often involves compiling data from several sources or separate sequencing runs, leading to the emergence of batch effects (BEs) (Leek *et al.* 2010). Batch effects are systematic differences between groups of samples that arise from variations in sample preparation and data generation processes. BE’s cause differences in the measurements between batches that are unrelated to biological variation (Lazar *et al.* 2012). Therefore, it is essential to take these effects into consideration before analysis.

Fortunately, there are several methods for Batch Effect correction (BEC) available. These methods can be roughly categorized into two groups: (i) methods that perform correction using linear combination of genes and (ii) methods that perform correction for each gene separately. Examples of methods that do gene-wise correction include ComBat (Johnson *et al.* 2007) and the removeBatchEffect, a function from the LIMMA R-package (Ritchie *et al.* 2015). Examples of methods that perform correction on the linear combination of genes include Harman (Oytam *et al.* 2016), Factor Adjustment method (FAbatch) (Hornung *et al.* 2016), and Surrogate Variable Analysis (SVA) (Leek and Storey 2007). The SVA method is used to correct for latent, unknown sources of unwanted variation rather than known batch effect variables. Furthermore, previous methods are designed to work for normalized continuous data, but methods have also been developed for raw count data of sequence reads. These methods include a variation on the empirical Bayes method ComBat-Seq (Zhang *et al.* 2020), and POIBM (Holmström *et al.* 2022). Finally, methods, like Seurat (Hao *et al.* 2022) and Harmony (Korsunsky *et al.* 2019), aim to correct BEs specifically in single-cell RNA-Seq data.

The large number of available BEC methods raises these questions: How do we select the best performing BEC method(s)? How do we rank different BEC methods against each other? These are fundamental questions for selection, comparison, and development of BEC methods, and we lack standardized methods of evaluating their performance. Many BEC method articles utilize visualizations with hierarchical clustering, principal component analysis (PCA) and/or multi-dimensional scaling (MDS) to show the presence and removal of BEs [e.g. Johnson *et al.* (2007), Pussila *et al.* (2018)]. Unfortunately, these visualizations become less informative when we compare numerous methods or when the differences in batch effect between BEC methods are subtle. Therefore, various metrics and statistics have been proposed for the comparison of BEC methods. Luo *et al.* (2010) evaluated classifier prediction accuracies from various processed and unprocessed datasets. Chen *et al.* (2011) predicted differentially expressed genes from artificial datasets and tested the accuracy of this process and the correlation between nominal and observed differential expression. In addition, Principal Variation Component Analysis (PVCA) has been used to identify the sources of variation from the data (Li *et al.* 2009, Chen *et al.* 2011). Another PCA-based evaluation method for BEs is guided-PCA (g-PCA) (Reese *et al.* 2013). Finally, we see evaluation metrics for evaluating batch correction for single cell RNA-seq datasets (Büttner *et al.* 2019).

**Figure 1:**
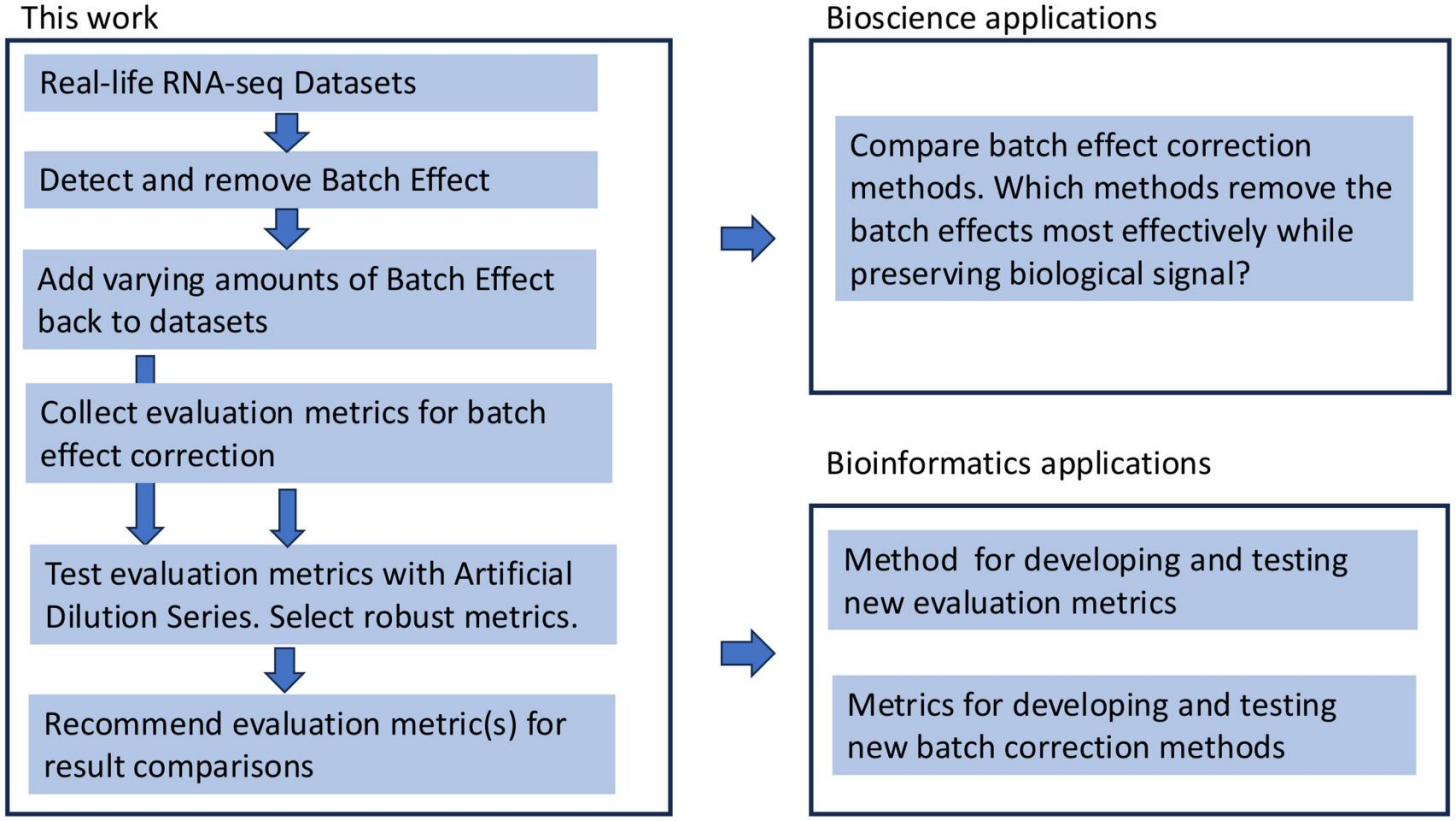
Project overview: this figure illustrates the main steps of our project, highlighting how our approach supports projects that require batch correction. Additionally, the selected metrics can be applied to other bioinformatics tasks, and the pipeline can be used to develop new evaluation metrics.

Given the availability of evaluation metrics, it remains unclear which ones should be used to evaluate BEC methods. To address this question, we conducted tests to evaluate the ability of various evaluation metrics to distinguish different levels of BEs, using the Artificial Dilution Series (ADS) approach. We gathered a collection of previously used and novel evaluation metrics for batch correction and examined their performance using datasets with varying amounts of batch effect noise. Each metric was scored based on its ability to discriminate between different noise levels. This test setup has previously been used when comparing classifier evaluation metrics (Plyusnin *et al.* 2019). Additionally, as BEC methods should preserve the biological signal while removing BEs, we also evaluated how well the biological signal was retained throughout the process. Overall, our analysis enables us to provide recommendations on which metrics should be used for comparison of BEC methods. See Fig. 1 for an outline of the project’s applications. Key points in this article are:

- We introduce a novel framework, ADS, for systematic comparison of BEC evaluation metrics.
- ADS can be applied to any dataset with a measurable batch signal, using any suitable BEC method and any set of evaluation metrics.
- We demonstrate notable differences in the performance between compared evaluation metrics.
- Results suggest top-performing metrics for both bulk RNA-Seq and scRNA-Seq BEC evaluation.
- We show that intermediate levels in the ADS series are critical for revealing differences in metric sensitivity.
- We evaluate metrics using both batch and biological groupings and demonstrate that usually the signal ratio provides the best measure for metrics’ performance.

## 2 Methods

### 2.1 Definitions

Batch effects: systematic differences in data between samples processed in different batches that are unrelated to any biological variation (Leek *et al.* 2010, Lazar *et al.* 2012). We assume that the BEs are (i) constant additive changes, shifts up or down, and/or (ii) multiplicative changes that affect the scale of the values within each batch. We further assume that these shift and scale changes are constant for each gene within each batch. These definitions are identical to the definitions used by Johnson *et al.* (2007).

Batch effect correction: methods of data normalization that attempt to estimate and remove these BEs from the data.

Biological effects: observed differences in gene activities between the biological sample groups. These are the signals that the research aims to find. In this article, we consider only shifts or additive changes in the biological effects. Furthermore, when we refer to a biological signal, we refer to differences in expression between sample groups indicative of the underlying biological effects.

Batch and biological sample groups: batch groups consist of samples processed under the same technical conditions, whereas biological sample groups consist of samples from the same biological condition.

Evaluation metrics: numerical measures of batch effect size and cluster separation. These metrics aim to measure the “well-mixedness” of BE corrected data.

Artificial dilution series: a method of generating series of test data with controlled levels of noise added. It has previously been used for evaluating gene ontology classifier metrics (Plyusnin *et al.* 2019). In this work, we use it to add controlled levels of BE to the data.

Mathematically, BEs can be defined as follows: the batch effect parameters are denoted as $\gamma_{i,g}$ and $\delta_{i,g}$, where γ is the location batch effect and δ is the multiplicative batch effect. Thus, the normalized expression value for sample *j*, batch *i* and gene *g* is written as:


$$Y_{g,i,j}=\alpha_{g}+X\beta_{g}+\gamma_{i,g}+\delta_{i,g}\varepsilon_{i,j,g}$$

where α is the baseline expression of the gene *g*, *X* is the design matrix of the sample conditions and β is the biological effect of interest (Johnson *et al.* 2007). ε is the normally distributed error term.

The data corrected for BEs are represented as


$$Z_{i,g,j}=\frac{1}{{\hat{\delta }}_{j,g}}(Y_{g,i,j}-{\hat{\alpha }}_{g}-X{\hat{\beta }}_{g}-{\hat{\gamma }}_{i,g})+{\hat{\alpha }}_{g}+X{\hat{\beta }}_{g}$$

where $\hat{\delta }$ and $\hat{\gamma }$ are the estimators of BEs (scale and shift), $\hat{\alpha }$ and $\hat{\beta }$ are the estimators of baseline expression and biological effects.

### 2.2 Datasets

We used four different real-life RNA-Seq datasets in our study, each with varying number of samples. Two of the datasets are of bulk RNA-Seq, and two single-cell RNA-Seq datasets. Each dataset has at least two batches and at least two biological groups of interest. We use one relatively large bulk dataset (in-house dataset), one smaller bulk dataset, along with two single-cell examples, one larger (thousands of cells) and one smaller (hundreds of cells). The number of samples/cells as well as batches and biological groups of interests are detailed in Table 1. We selected large and small datasets to review if metrics have problems with different data types.

**Table 1:** The summary of datasets used in this study with the number of samples/cells, sample/cell types (biological groups), and batches.

Dataset	RNA-Seq	Samples/cells	Batches	Sample/cell types
In-house colon	Bulk	107	4	5
HeLa Kyoto	Bulk	10	2	2
mESC	Single-cell	469	2	3
Pancreas	Single-cell	6321	4	13

### 2.3 In-house cancer data

The first dataset evaluated is our larger in-house RNA-seq collection, used in the study of mitotic abnormalities in Lynch-syndrome–affected colon mucosa by Pussila *et al.* (2024). The dataset includes samples from human patients representing normal colon tissue, benign growths, and malignant growths (cancer). The patients fall into two groups: individuals with an inherited increased risk of cancer and those without.

The normal tissue samples are further subdivided into three categories based on observations at the time of sampling: (i) without growth, (ii) with benign growth, and (iii) with malignant growth. These classifications are based on visual inspection during sampling and, for samples containing abnormal growth, subsequent histological confirmation. Biologically, we expect samples from normal tissue to separate from those with benign or malignant growth.

The in-house dataset exhibits two separate BEs. The first arises from the use of two sample preservation techniques, liquid nitrogen ($N_{2}$) and RNA-later reagent, during sample preparation. The second results from two distinct sequencing runs (“New” and “Old”), with the newer run enriched for samples with low read counts. Several biological sample groups differ in size and are unevenly distributed across these batches, making this dataset a challenging case in which multiple BEs may be confounded with biological factors.

### 2.4 HeLa Kyoto cell line data

Another smaller dataset used here was the HeLa Kyoto cell line dataset used by Procida *et al.* (2021). This RNA-Seq dataset is not associated with disease, but instead studies the expression of the JAZF1 protein. The dataset contains 10 samples from two batches (4 and 6 samples in each batch, respectively), which introduce a well-defined batch effect which makes this dataset suitable for comparison. Figure 1, available as supplementary data at *Bioinformatics Advances* online, shows multi-dimensional scaling plots that visualize the amount of BEs in the two bulk RNA-Seq datasets used. This dataset is available in NCBI Gene Expression Omnibus (GEO) (Edgar *et al.* 2002), with the GEO Series accession numbers GSE163214 and GSE163318.

### 2.5 mESC single-cell data

BEs are a persistent issue in single-cell RNA-Seq (scRNA-Seq) experiments, and correcting them is a difficult problem to evaluate (Chazarra-Gil *et al.* 2021, Antonsson and Melsted 2025). Therefore, we also compare the performance of the BE evaluation metrics in single cell RNA-Seq data from mouse embryonic stem cell (ESC) cultures, cultured under three different conditions, serum/LIF and 2i/LIF and a2i/LIF (Kolodziejczyk *et al.* 2015). The two different batches are the two replicates from each of the three conditions. The dataset has a total of 704 cells. The raw count data was downloaded from the Espresso database (https://espresso.teichlab.sanger.ac.uk/).

### 2.6 Single-cell pancreas dataset

Another single-cell dataset included in the comparison is a single-cell RNA-seq dataset from pancreatic islet cells, provided by https://satijalab.org (Hao *et al.* 2022). The dataset consists of 6321 cells, across 13 cell types with BEs arising from four different technologies used to produce it; CelSeq, CelSeq2, Fluidigm C1, and SMART-Seq2. This dataset serves as an example of a larger scRNA-Seq dataset of several thousand cells.

### 2.7 Data normalization and pre-processing

The bulk RNA-Seq datasets were normalized using the DESeq2 R-package (Love *et al.* 2014). Variance Stabilizing Transformation (VST), from the same package was also applied. This transformation removes some of the unreliable variance among the low-count observations. These are usually less reliable than higher count observations.

The single-cell datasets were normalized and log-transformed using the R-package “scuttle” (McCarthy *et al.* 2017), using the logNormCounts function.

### 2.8 Batch effect correction

The BEs in the bulk RNA-Seq datasets were corrected using ComBat (Johnson *et al.* 2007), and in the single-cell datasets using the Harmony-method (Korsunsky *et al.* 2019). Note that Harmony computes batch-correction on data transformed by PCA, so no further PCA-transformations are necessary in this case. These methods provide an estimate of the batch effect size, used to generate the ADS.

### 2.9 Evaluation metrics

We collected and developed a number of metrics to evaluate the effectiveness of BEC. A good metric should naturally monitor the amount of BE in the data. In addition, we argue that it should monitor the amount of biological signal in the data. This ensures that the BEC used is not simply removing all the signals from the data. Therefore, we tested most of the metrics (i) by evaluating the separation of batch groups, (ii) by evaluating the separation of biological sample groups, and (iii) by evaluating the ratio between the separation of batch groups and separation of biological groups. Our aim is to look for metrics that show good correlation with the embedded batch signal in the dataset.

Notice that the evaluation metrics used come from very different backgrounds. Some are based on standard statistics (ANOVA test), some come from cluster result evaluation, and some are specifically designed for evaluation of BEC methods. Table 2 summarizes the metrics used along with their short descriptions.

**Table 2:** The summary of evaluation metrics used in this comparison.

	Abbreviation	Description	Citation
F-score	F-score	Calculates ratio of between-group variation and within-group variation, scaled genewise	This work
Davies–Bouldin index	DB-index	Cluster separation metric, compares between-group and within-group scatter measures	This work
k-Nearest neighbors	kNN	Calculates mean proportion of k-nearest neighbors, that belong to the same sample group.	This work
Kullback–Leibler distance	KLdist	Estimates the distributions of sample groups and measures distance between those distributions.	Hornung *et al.* (2016)
Minimum distance	mindist	Calculates the minimum distance between sample groups	This work
Guided principal components analysis	gPCA	A ratio of “guided” PCA and “normal” PCA	Reese *et al.* (2013)
Principal variance components analysis	PVCA	Uses principal components regressed on sources of variation (batch/biological groups) to calculate a proportion of variance	Hornung *et al.* (2016)
kBET	kBET	A $\chi^{2}$ based test that determines, whether random neighbourhoods are well mixed.	Reese *et al.* (2013)
CellMixS	CMS	A knn-based cell-specific mixing score for single-cell data	Lütge *et al.* (2021)

### 2.10 Distance measure

For most of these metrics, we first calculate the pairwise distance between samples, and collect them into a distance matrix *D*. We used Pearson’s correlation distance measure. Here, the distance between samples *i* and *j* is defined as


$$D_{i,j}=1-\rho_{X_{i},X_{j}},$$

where $\rho_{X_{i},X_{j}}$ is the Pearson correlation between sample *i* and *j*. Notice that other distance measures, such as the cosine distance or Euclidean distance, could be equally used in our system. We limit our analysis to Pearson’s correlation distance for simplicity.

All distance measures were calculated from the VST-transformed or log-count data. For larger datasets, mainly the pancreas single-cell data, PCA was used to reduce dimensions before computing the distance matrix, to reduce computation time.

### 2.11 ANOVA F-statistic

The first metric used to evaluate batch correction is derived from the ANOVA F-statistic. It is defined as:


$$F=\frac{\sum_{(i,j)\in C_{0}}D_{i,j}}{\sum_{k=1}^{K}\sum_{(i,j)\in C_{k}}D_{i,j}}.$$

where *K* is the number of clusters (BEs or biological groups of interest), *D* is the distance matrix of the data, $C_{k}$ contains the indices of the *k*: th cluster and $C_{0}$ contains the index pairs that belong to different clusters. Lower values of this metric indicate better mixing of the clusters, and higher scores indicate more cluster separation. We also developed a “scaled” version of the F-statistic, defined as:


$$F_{\mathrm{scaled}}=\frac{\sum_{(i,j)\in C_{0}}D_{i,j}/Q_{i}(p)}{\sum_{k=1}^{K}\sum_{(i,j)\in C_{k}}D_{i,j}/Q_{i}(p)},$$

Here, we add a scaling parameter $Q_{i}(p)$ to account for cluster size, which is defined as the *p*-quantile of the *i*: th column of the distance matrix.

### 2.12 Davies–Bouldin cluster separation metric

Another used metric is the Davies–Bouldin index (Davies and Bouldin 1979). It is mainly used to evaluate clusters against a gold-standard grouping of data points. It is defined as:


$$\mathrm{DBI}=N\sum_{i=1}^{N}\max_{i\neq j}(\frac{S_{i}+S_{j}}{M_{i,j}})$$

where $S_{i}$ and $S_{j}$ are within-cluster distance measures of clusters *i* and *j* (mean distance from each cluster’s centroid), and $M_{i,j}$ a distance measure between clusters *i* and *j*. The distance used here is the Pearson distance. The distance between the clusters is defined as the mean of the distances of the samples between the clusters.

### 2.13 K-nearest neighbors

A kNN-based approach was also used (kNNprop), similar to a “mixture score” presented in (Lazar *et al*. 2012), a very simple metric that computes the mean proportion of each data points’ k-nearest neighbors that belong to the same cluster/batch group, and averages them over every group, i.e.


$$P_{k}=\frac{1}{K}\sum_{j=1}^{K}\left(\frac{1}{k}\sum_{x\in \mathrm{kNN}}\frac{\left|\{x\in C_{j}\}\right|}{\left|C_{j}\right|}\right),$$

where *k* is chosen as the mean batch cluster size.

### 2.14 Minimum distance

The mindist metric simply computes the mean minimum distance between each cluster. The metric is computed as


$$\frac{2}{K(K-1)}\sum_{i}^{K}\sum_{j\neq i}^{K}\min D(C_{i},C_{j})$$

where *K* is the total number of clusters and $C_{i}$  $C_{j}$ are the *i*: th and *j*: th clusters, respectively. mindist is here as a baseline metric that we expect to show weaker performance than other metrics.

### 2.15 PCA-based metrics

One of the metrics used is a g-PCA metric presented in Reese *et al.* (2013), primarily used for detecting BEs. The metric is defined as the ratio of variances from the first principal components of “guided” and “unguided” principal component analyses. The quantity used in comparison is the percentage of the total variation explained by the batch variable. Another PCA-based metric is pvca from the R package bapred. It was used by Hornung *et al.* (2016) to evaluate batch correction.

### 2.16 Kullback–Leibler divergence

We also used the metric kldist (Hornung *et al.* 2016), which calculates a distance measure based on the Kullback–Leibler divergence of the estimated densities between the pairwise distances of samples in each batch and sample group. It is calculated as in the R package bapred (Hornung *et al.* 2016).

### 2.17 Single cell metrics

Several evaluation metrics have been proposed for measurement of BEs in scRNA-Seq data specifically, including kBET (Büttner *et al.* 2019), cKBET (Zhao *et al*. 2023), CellMixScore (Lütge *et al.* 2021), RBET (Hu *et al.* 2025), etc. We selected two metrics specifically for measuring BEs in single cell data, in addition to the previously introduced metrics.

### 2.18 kBET

This test metric was developed to detect BEs in single cell RNA-Seq data (scRNA-Seq) (Büttner *et al.* 2019). We evaluated the performance of this metric compared to the others in the mESC single-cell dataset. As this metric is developed for detecting BEs specifically in scRNA-Seq data, we excluded it from the comparison in the bulk RNA-Seq datasets. We used the rejection rate given by kBET as a measure of BEs.

### 2.19 Cell mix score

Another metric used for detection of BEs in scRNA-Seq included in this comparison is the Cell Mix Score (CMS) (Lütge *et al.* 2021). A KNN-based metric, CMS compares batch-specific distance distributions of each cell’s *k*-nearest neighbors. It is implemented using the R-package CellMixS. The original CMS function computes the mixing score for each cell, here we use the average over all cells/samples to represent the total mixing score on the whole dataset.

### 2.20 Methods

We wanted to create a testing method for evaluation metrics to see how they detect varying amounts of BE in the dataset. We used an approach called the ADS (Plyusnin *et al.* 2019) to create these test datasets. Here, we use a real life RNA-seq dataset for ADS process. We alter this data, either by adding noise to it, or by removing the biological signal from it. Furthermore, we vary the way how we add small variance to datasets with the same amount of noise or biological signal. In the following, we explain the methods that we use for these steps.

### 2.21 Artificial dilution series by adding batch effect

In this study we utilized actual BEs extracted from the data as noise. First, we fully removed BEs from the data using a selected BEC method, such as ComBat or Harmony, to obtain an estimate of the batch-corrected data. The estimated batch-corrected data were then used in the ADS approach and a series of datasets was constructed using the following formula:


$$ADS_{p}(Y_{i,g,j},Z_{i,g,j})=(1-p)Y_{i,g,j}+pZ_{i,g,j},$$

where $Y_{i,g,j},Z_{i,g,j}$ are the uncorrected and batch-corrected data respectively, for gene *g*, sample/cell *j* and batch *i*. Here, *p* takes values from 0 to 1 with increments of 0.1, representing the datasets with 0%, 10%, 20%, …, 100% of BEs removed. The metrics from previous chapters are then evaluated on each dataset in the ADS to assess their performance under varying levels of BEs. Text, available as supplementary data at *Bioinformatics Advances* online, shows a visualization of batch effect removal at three levels, showing the amount of BEs present in the uncorrected data, 50% corrected data and the fully corrected data.

### 2.22 ADS by removing the biological signal

The first ADS version allows for measuring the sensitivity of metrics to BEs. We developed a second ADS that varies the amount of biological variation in the data and the separation of biological groups of interest. This was done to ensure that the evaluation metrics respond also to changes in the biological signal, in case a batch correction method over-corrects for biological information outside the batch factor. To achieve this, we modified the ADS by removing biological variation from a dataset without BEs (e.g. batch-corrected data). We gradually reduced 0%, 10%, 20%, …, 100% of the mean from each biological group of interest. As a result, we obtained a series of datasets, ranging from those with properly separated biological groups to those with mixed biological groups. Figure 3, available as supplementary data at *Bioinformatics Advances* online, shows a visualization for this process.

### 2.23 Adding variance to datasets

ADS generates multiple datasets with the same amount of noise. These datasets, although they represent the same amount of signal, should show some variance from each other. This smaller variance aims to represent the type of variance that we expect to see between different datasets with the same amount of signal. We use this to measure the sensitivity of each metric to this smaller variance and look at the resulting variance within each signal level of ADS datasets. Below we describe two methods for this.

### 2.24 Dirichlet-multinomial resampling of gene counts

The first variance-adding algorithm uses a statistical model to generate artificial technical replicates for each sample in RNA-seq data. These are then used to create datasets with small variance. This algorithm uses a multinomial distribution on each column (sample) of the count dataset, using the original count data to define the probabilities of each gene in the sample. A similar method has been used before in the analysis of differential expression in RNA-seq data [see, e.g. Al Seesi *et al.* (2014)]. Our model assumes a Dirichlet-multinomial distribution on the count dataset, using the columns of the original count data matrix as hyperpriors.


$$\begin{array}{c} x_{i}∼\;\text{Multinomial}(n_{i},\theta_{1}^{\left(i\right)},\ldots ,\theta_{k}^{\left(i\right)})\\ \theta_{i}=(\theta_{1}^{\left(i\right)},\ldots ,\theta_{k}^{\left(i\right)})∼\text{Dirichlet}\;(\alpha_{i}). \end{array}$$

Here, the hyperparameter vector $\alpha_{i}=(\alpha_{1}^{\left(i\right)},\ldots ,\alpha_{k}^{\left(i\right)})$ contains the original raw counts for a column *i* in the data. The BEs of the original data are retained on this resampled dataset. Batch correction is then performed on this resampled dataset, correction parameters are extracted and then applied on each level of p=0,10%,…,100% as described before. The metrics *M* are then evaluated with each dataset.

Algorithm 5.1
*Dirichlet-Multinomial resampling of raw count data and batch correction evaluation. Let* $n_{\mathrm{iter}}$  *denote the number of iterations. For i=1,…*, $n_{\mathrm{iter}}$*, do*

1. For each column *j* in the raw count data, sample $x_{j}∼\text{Dirichlet-Multinomial}(n_{j},\alpha_{1},\ldots ,\alpha_{k})$

2. Construct a new dataset $X_{\mathrm{count}}^{\left(i\right)}$.

3. Compute normalization (DESeq2 or scuttle) and log-transformation on $X_{\mathrm{count}}^{\left(i\right)}$ to produce $X^{\left(i\right)}$

4. Optional: Compute PCA on $X^{\left(i\right)}$. Use first $n_{PC}$ PCs.

5. Estimate batch-corrected data $Z^{\left(i\right)}$, using ComBat or Harmony.

6. Construct an ADS of p=0,10%,…,100% levels

$\mathrm{ADS}(X^{\left(i\right)},Z^{\left(i\right)})=X_{p}^{\left(i\right)}$

7. Compute $M_{\mathrm{batch}}(X_{p}^{\left(i\right)})$, $M_{\mathrm{bio}}(X_{p}^{\left(i\right)})$ and $M_{\mathrm{ratio}}(X_{p}^{\left(i\right)})=M_{\mathrm{batch}}(X_{p}^{\left(i\right)})/M_{\mathrm{bio}}(X_{p}^{\left(i\right)})$ for each value of *p*, and save in a list.

### 2.25 Bootstrap

Another way to add variance to generated datasets is by reselecting the variables, genes, to distance calculus. Here we use the standard bootstrapping to resample genes to distance calculus. This can omit some genes and include some genes more than once. The process is repeated $n_{\mathrm{iter}}$ times.

Algorithm 5.2 *Gene bootstrapping of batch corrected dataset. For each level of p, and for each metric M, repeat* $n_{\mathrm{iter}}$  *times*

1. Sample new dataset $X_{\mathrm{new}}$ with replacement from $X_{p}$

2. Optional: Compute PCA on $X_{\mathrm{new}}$. Use first $n_{PC}$ PCs.

3. Compute $M_{\mathrm{batch}}(X)$, $M_{\mathrm{bio}}(X)$ and $M_{\mathrm{ratio}}(X)=M_{\mathrm{batch}}(X)/M_{\mathrm{bio}}(X)$ and save in a list

This method tests the stability of the results, as the genes used in the calculus are varied.

### 2.26 Alternatives for Dirichlet-multinomial

The Dirichlet-multinomial model has certain limitations, when it comes to estimating sparsity, especially in single cell data. Zero-inflated models are commonly used in the context of scRNA-Seq data simulation, such as Splatter (Zappia *et al.* 2017) and SymSim (Zhang *et al.* 2019). However, in our case these methods would have been unwieldy for the purpose of generating variance on an existing data, since they cannot estimate the BEs and biological signal directly. We deemed the Dirichlet-multinomial model sufficient in generating variance around an existing dataset, since we have no need to generate entirely new data. Text, available as supplementary data at *Bioinformatics Advances* online, discusses the alternatives more.

### 2.27 Visualizing and scoring evaluation metrics with ADS

**Figure 2:**
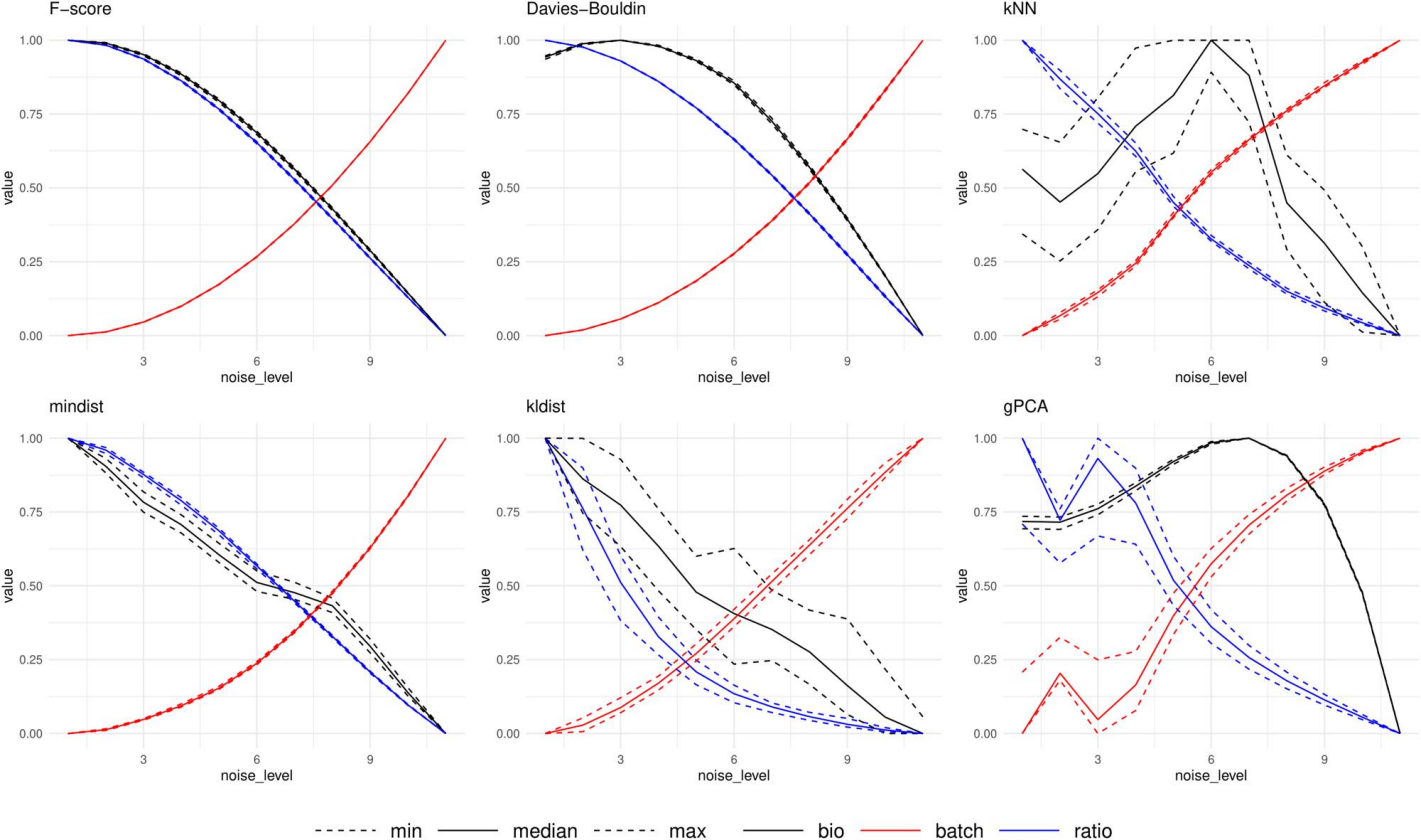
The results of the performance metrics on the multinomial resampled datasets of the in-house colon cancer data. For each graph, three graphs are shown for the biological signal, batch effect signal and the ratio of biological and batch effects. These are shown against the noise level, the amount of batch effect noise in the ADS (fully corrected data on the left, uncorrected data on the right.) Notice how intermediate noise levels show unstable behavior and/or larger scattering for some metrics.

First, the results from ADS can be visually evaluated. We define the noise level here as the amount of alteration performed on the data either by adding the BE in the data or by removing the biological signal from the data. Here, we (i) group the datasets from each noise level to their own group, (ii) order these groups with their noise levels, (iii) calculate an evaluation metric score for each dataset within each group, and (iv) generate a box plot visualization for each noise level. The generated visualization (see Fig. 2 and Text, available as supplementary data at *Bioinformatics Advances* online) will quickly show if the tested metric is able to separate the different noise levels from each other.

Visual evaluation is an impractical solution when we compare several evaluation metrics. Here, a table representation with a selected scoring is a more suitable way for comparison. A good evaluation metric should perform so that $M_{1}>M_{2}>M_{3}\ldots >M_{11}$. This suggests that we should use either linear or rank correlation, between the noise level and generated metric scores, as a measure of correlation. As the correlation is often non-linear, we selected rank correlation as a scoring function for our ADS results.

### 2.28 Spearman’s rank correlation

As a numerical measure of the metrics’ correspondence to the ADS levels, we used Spearman’s rank correlation. Rank correlation is computed by pooling all results for each metric and ADS level, and computing Spearman’s rank correlation with the corresponding rank from 1 to 11, representing the ADS datasets.

## 3 Results

For each dataset, we repeated $n_{\mathrm{iter}}=50$ iterations of the process described in Algorithm 5.1. We also repeated 50 iterations of the Algorithm 5.2. (bootstrap) for the bulk RNA-Seq datasets only. For the single-cell datasets, we use PCA with the first 50 principal components. We present the median, minima and maxima of each iteration and ADS level in figure format and the rank correlations in table format.

In addition, we repeated the above process on the bulk RNA-Seq datasets, using the diluted biological signal variation of the ADS, described in Section 2. The results can be seen in Fig. 4, available as supplementary data at *Bioinformatics Advances* online, and Tables 1 and 2, available as supplementary data at *Bioinformatics Advances* online.

### 3.1 Visual inspection of selected evaluation metrics

Our initial examination of ADS results involves a visual inspection. The first set of results, shown in Fig. 2, illustrates the outcomes when we manipulate the batch effect using ADS, on our in-house cancer data. We present three distinct visualizations of ADS results for each metric:

- The black visualization demonstrates how well the metric distinguishes between ADS groups while using biological sample labels.
- The red visualization showcases the metric’s ability to differentiate between the batch groups at different levels of the ADS.
- The blue visualization represents the ratio between the metric measuring the separation of biological groups and batch groups.

**Figure 3:**
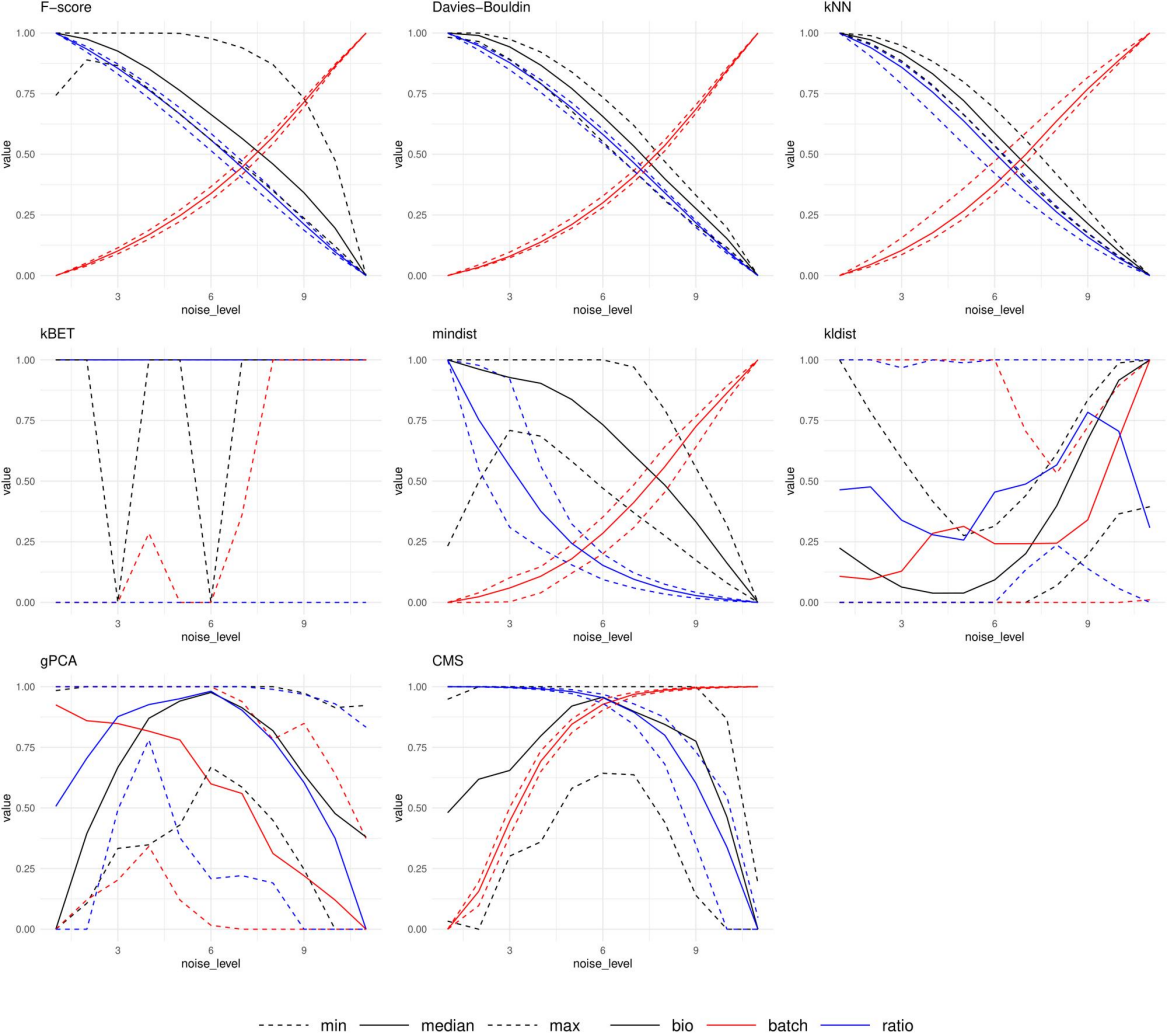
The results of the performance metrics on the multinomial resampled datasets of the pancreas single-cell data. The test metrics kBET and CMS were included in this comparison, as they were developed specifically for single-cell datasets. Notice the weaker performance of kBET, kldist, and gPCA.

In Figs 2 and 3, we show which metrics and test systems produce the most pronounced separation across various noise levels, for our in-house RNA-Seq data and the single-cell pancreas data. Figures 5 and 6, available as supplementary data at *Bioinformatics Advances* online, show results for the other two datasets. Our expectation is to observe a clear decreasing trend in biological signals and in the ratio of the two signals, and a noticeable increase in batch-related signal. Each score has been standardized by the minimum and maximum across the levels in the visualization.

The results of Figs 2 and 3 show that in our datasets, the F-score and Davies–Bouldin index showed the most stability across the ADS levels, while kNN and mindist showed good results in some datasets, while mixed results on others. Results of metrics like kldist and gPCA showed more unstable results. Single-cell metrics results are shown in 3, where the kBET metric fails to detect BEs, while the CellMixS (CMS) metric shows relatively good performance. In our comparison, simple distance-based metrics, such as F-score and Davies–Bouldin index, showed the best performance in measuring BEs.

Text, available as supplementary data at *Bioinformatics Advances* online, also includes the same results from ADS with reducing biological signal (see Fig. 4, available as supplementary data at *Bioinformatics Advances* online). The figure shows good results for F-score and Davies–Bouldin, weaker result for kNN and very weak results for mindist, kldist, and gPCA.

### 3.2 Rank correlation results of the compared evaluation metrics

We present Spearman rank correlation results for the bulk RNA-Seq datasets in Table 3, and the scRNA-Seq data in Table 4. Metrics that detect changes in batch effect levels in the ADS show a higher rank correlation. We present the results for each metrics ability to detect changes in (i) biological sample groups, (ii) batch sample groups, and (iii) signal ratio of A and B, as the level of batch effect changes in the ADS. The best ranking metrics for both bulk and single-cell RNA-Seq can be found in Table 5.

**Table 3:** The rank correlation scores (Spearman’s rho) across the bulk RNA-seq datasets for all types of signal with two variance models, where ADS was generated using different levels of batch signal.

Variance model	Data	Signal	Metrics					
			F-score	DB-index	kNN	mindist	kldist	gPCA
Resampling	Inhouse	Bio	0.969	0.896	0.482	**0.978**	0.976	0.232
		Batch	0.996	0.996	**0.996**	0.993	0.996	0.963
		Ratio	0.994	0.996	**0.996**	0.996	0.996	0.965
	HeLa Kyoto	Bio	**0.996**	**0.996**	0.967	−0.872	0.697	0.606
		Batch	**0.996**	**0.996**	0.981	0.995	0.931	−0.605
		Ratio	**0.996**	**0.996**	0.985	0.988	0.898	0.946
Bootstrap	Inhouse	Bio	**0.98**	0.98	0.795	−0.423	0.975	−0.335
		Batch	0.994	**0.996**	0.995	0.995	0.995	0.88
		Ratio	0.992	**0.996**	0.996	0.994	0.995	0.89
	HeLa Kyoto	Bio	**0.995**	**0.995**	0.964	−0.727	0.428	0.698
		Batch	**0.996**	0.994	0.972	0.995	0.918	0.698
		Ratio	**0.996**	0.995	0.98	0.979	0.871	0.873

The best performing metrics are marked with bolded values.

**Table 4:** The rank correlation scores (Spearman’s rho) for the single-cell datasets.

Variance model	Data	Signal	Metrics							
			F-score	DB-index	kNN	kBET	mindist	kldist	gPCA	CMS
Resampling	mESC	Bio	0.845	0.875	**0.942**	−0.183	0.838	0.798	0.761	0.363
		Batch	0.985	**0.986**	0.975	0.503	0.958	−0.277	0.271	0.951
		Ratio	**0.967**	0.963	0.962	0.502	0.962	0.255	0.732	0.848
Resampling	Pancreas	Bio	0.774	0.924	**0.978**	−0.029	0.794	−0.628	0.064	0.154
		Batch	0.985	0.99	0.987	0.277	0.992	0.159	−0.742	**0.995**
		Ratio	0.987	0.992	0.988	0.264	0.99	−0.029	0.265	**0.994**

The best performing metrics are marked with bolded values.

**Table 5:** The mean ranks of each metric across all RNA-seq datasets, for the ADS applied to batch effects.^a^

Data	F-score	DB-index	kNN score	kBET	mindist	kldist	gPCA	CMS
Bulk	2.458	**1.958**	3.167	NA	3.417	4.167	5.833	NA
Single-cell	4.333	**2.500**	3.333	7.167	**2.500**	6.167	5.500	4.500

a The best ranking metric marked with bolding.

The results show that some of the metrics are more stable than others in measuring the change in batch and biological factors. The F-score, Davies–Bouldin index, and kNN show the most stability in measuring the level of BEs and biological effects across all levels of the ADS, across most datasets. In particular, the Davies–Bouldin index showed the best performance in our comparisons. These metrics showed stable results on both bulk and single-cell RNA-Seq datasets (Tables 3 and 4). More complicated metrics, such as gPCA showed the greatest degree of instability in measuring the desired effects. Therefore, for these datasets, we can infer the best metrics to measure the success of batch correction. This process can be repeated for other datasets.

### 3.3 Results on single-cell data

Results on the two single-cell datasets show a few interesting details. The metrics that performed the best in bulk RNA-Seq data seem to perform fairly well in single-cell data. The CellMixS (CMS) metric also performed quite well, especially on the larger single-cell pancreas data, where it showed the best performance. On the other hand, kBET, another metric developed specifically for single-cell data, performed poorly. In the case of the pancreas dataset, it showed a near uniform value of one across all resampled datasets and noise levels. In addition, the kNN-metric performed the best for measuring the biological signal on both single-cell datasets.

The metrics that showed the poorest performance in the bulk RNA-Seq data showed even poorer results in the scRNA-Seq data, as seen in Table 4 and Fig. 3. Notice, especially, the instability in the intermediate levels of the ADS, where the metric fails to detect the changes in the dataset.

### 3.4 Differences in results between bulk and single-cell data

In Fig. 3 we observe a considerably higher variance in the results on single-cell data compared to the results on bulk data. In the smaller bulk datasets, the minima and the maxima of the results stay fairly close to the median, while the gap is wider in the larger scRNA-Seq datasets. This may be due to both the increase in data points as well as the different batch correction methods used. We also observed that the bootstrap method is unsuitable for generating variance in sparse single-cell data, as the number of rows with zero counts is higher. Therefore, we omit the bootstrap results in the single-cell datasets.

## 4 Discussion

Having a reliable and well-functioning evaluation metric is critical for any selection task. The selection and evaluation of batch correction methods is not something that is often discussed in the current literature and is a topic that is often overlooked. Therefore, we wanted to develop a test platform that can be used to compare different evaluation metrics used in the BEC against each other. Our test platform, the ADS, generates multiple copies of selected RNA-seq data, while adding different amounts of batch effect to the created datasets. Such testing platform has not been previously developed.

Our results show consistency across several different datasets, across bulk and single-cell RNA-Seq, although some variation is seen. Theoretically, the ADS platform is applicable to any datasets where the noise signal is clearly defined. In our case, we used an estimate of the corrected data using BEC tools, ComBat and Harmony, to extract the noisy signal from the data. Then, intermediate steps between the “noisy” and “correct” data can be used to measure the ability of an evaluation metric to detect that noise. This platform can thus generalize beyond the RNA-Seq or batch effect correction context.

In the results from the tested datasets, we see that pairwise distance-based metrics mostly outperform local distance-based metrics. This seems contradictory to current understanding of best performing metrics for batch effect evaluation, especially in single-cell data. Recent work (Rautenstrauch and Ohler 2025) has shown that Silhouette-based metrics can be misleading for single-cell integration benchmarking, particularly when assumptions about cluster geometry are violated and when nested BEs are present. Instead, mixed use of both local and global distance-based metrics is recommended. Especially the Silhouette score, a measure similar to the Davies–Bouldin index, is criticized. An alternative to Silhouette, Batch Relative Area under the Silhouette curve (BRAS) has been proposed (Rautenstrauch and Ohler 2025), which uses all pairwise distances, similarly to our use of the F-statistic. However, DB-index and F-statistic showed almost equally good performance in our analysis with sc-RNA data. It is worth noting that local distance-based methods rely on nearest-neighbor relationships. Local neighborhoods can remain relatively similar even as global structure improves in ADS protocol, potentially reducing their sensitivity to gradual changes.

These findings should be interpreted in the context of the present study. The analysis is based on a tested set of bulk- and scRNA-seq datasets and may not capture the full range of all experimental conditions and batch structures. Metric performance may also depend on preprocessing choices and distance representations. These considerations highlight opportunities for further work. Extending the analysis across additional datasets and evaluation settings will help clarify the generality of these observations and refine best practices for assessing batch correction.

## Supplementary Material

vbag142_Supplementary_Data

## Data Availability

The data underlying this article are available in our GitHub repository, https://github.com/Aleksi95/BatchMetrics (in-house data). The other datasets are available in NCBI Gene Expression Omnibus (HeLa Kyoto cell line data, accession numbers GSE163214 and GSE163318), https://espresso.teichlab.sanger.ac.uk/ (mESC single-cell data) and https://satijalab.org/ (single-cell pancreas data).
