# Supplementary material for: Development and comparison of evaluation metrics for batch correction reveals performance differences
Source: Bioinform Adv. 2026 May 21;6(1):vbag142. doi: 10.1093/bioadv/vbag142 (PMC13221981; doi:10.1093/bioadv/vbag142)
Supplement: vbag142_Supplementary_Data [file vbag142_supplementary_data.zip › Supplementary_text.pdf]

Supplementary material: Development and Comparison of Evaluation Metrics for Batch Correction Reveals Performance Differences

Aleksi Laiho[1] Marjaana Laitinen[1] Liisa Holm[1] Petri Törönen[1]

[1]University of Helsinki

Corresponding author: aleksi.laiho@helsinki.fi

Abstract

This is the supplementary text for the article Development and Comparison of Evaluation Metrics for Batch Correction Reveals Performance Differences. The text discusses the differences of results in bulk and scRNA-Seq datasets, as well as alternative models for the Dirichlet-Multinomial noise generation.The text also includes supplementary figures that show the batch effects of the bulk-RNA datasets used. In addition, we show the line plot results from the ADS analysis of the datasets, where a plot was not included in the main article, as well as results from ADS with diluted biological signal for our in-house data.

1 Supplementary text

1.1 Results on single-cell data

Our results show consistent performance across different metrics on both single-cell and bulk RNA-Seq data. For example, in both cases the F-score and Davies-Bouldin index metrics showed good and consistent performance in the ADS datasets. However, a surprising result shows the kBET metric showing poor performance in the ADS generated on the single-cell dataset. The result is especially unexpected as the metric is specifically developed to detect batch effects in single-cell RNA-Seq data. The metrics that showed poor performance in the bulk RNA-Seq data also showed poor results in the scRNA-Seq data.

This work is shared under a CC BY-SA 4.0 license unless otherwise noted

1.2 Negative binomial modeling of RNA-Seq data

In our article, we used the Dirichlet-Multinomial model to sample count data from the distribution estimated from an existing data, to provide variation to datasets in the ADS. We used the same model across all datasets, both bulk and single-cell data. We are aware of the limitations to this model, especially in modeling sparse scRNA-Seq data, but in the general purpose of generating variation around the original data points, we found it serviceable.

Another model commonly used to model RNA-Seq data is the Negative Binomial model. Given the gene- and sample-wise mean parameter $\mu_{j,g}$ and the gene-wise dispersion parameter δ_g, that can be estimated using the DESeq2 package, the individual read counts for sample j and gene g follow the Negative Binomial distribution

$$C_{j,g} \sim NB(\mu_{j,g}, \delta_g).$$

This model is provided as an alternative model for bulk RNA-Seq modeling in our BatchMetrics R-package. For scRNA-Seq modeling, a zero-inflated model is commonly used, but not implemented here.

2 Supplementary figures

We show Multi-Dimensional Scaling (MDS) plots (see Fig.1) that show the biological signals and the batch effects of the datasets used.

2.1 Supplementary figures of Artificial Dilution Series process

Here, we show MDS plots in Fig.2 that visualize the effect of batch effect removal on the in-house dataset. We also show MDS plots in Fig.3 that visualize how removal of the biological signal affects the in-house dataset.

This work is shared under a CC BY-SA 4.0 license unless otherwise noted

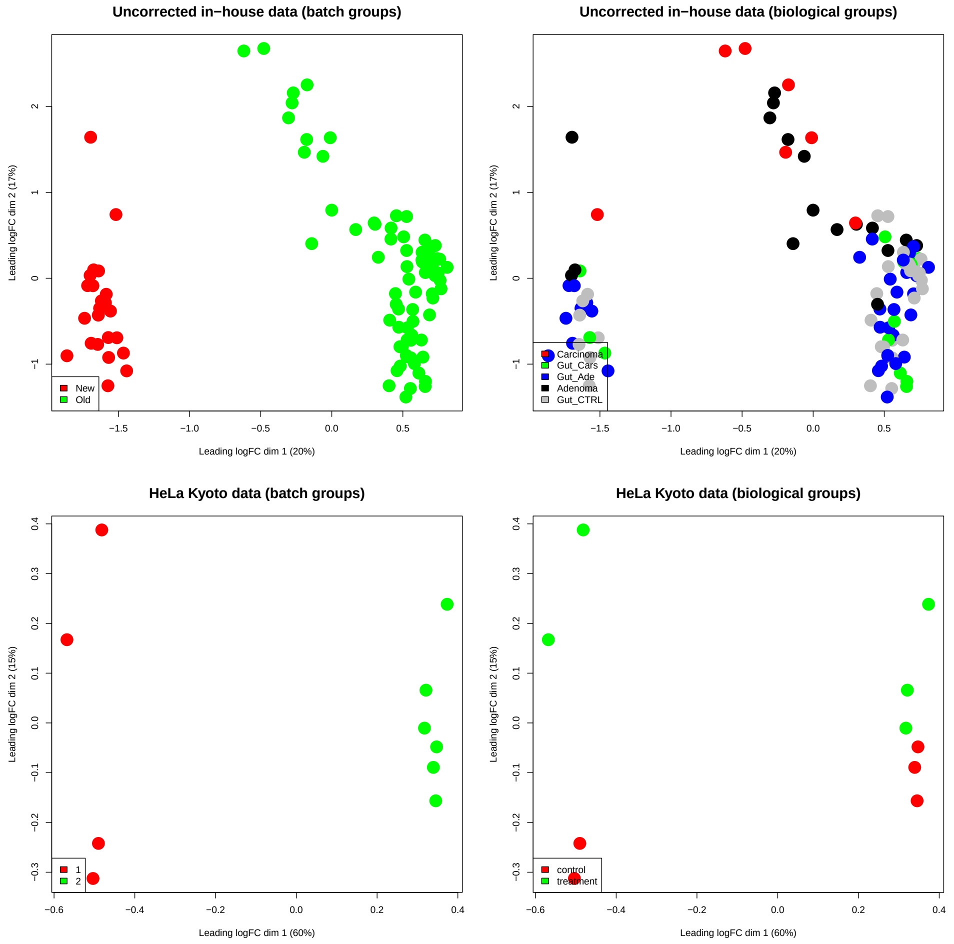

Figure 1: The batch effects, visualized with Multi-Dimensional scaling (MDS) on the in-house colon dataset (upper row) and the HeLa Kyoto cell line dataset (lower row). The first column shows the batch effects and the second column shows biological signals. Both datasets represent a strong batch effect.

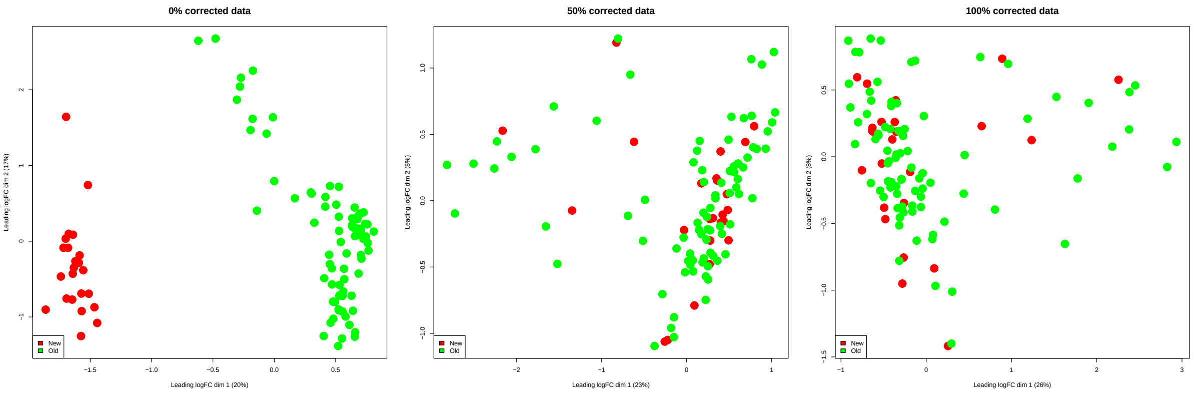

Figure 2: Multidimensional scaling (MDS) - plot of three stages of the ADS, raw data with no correction, 50% of the correction and the full batch corrected dataset.

This work is shared under a CC BY-SA 4.0 license unless otherwise noted

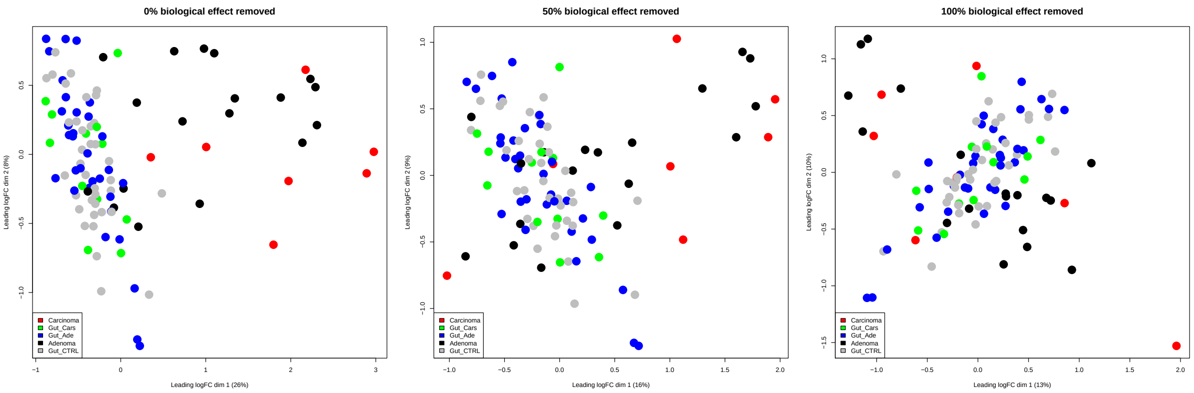

Figure 3: The 0%, 50% and 100% levels of biological variation removed from the batch-corrected data. The mean of each biological group is subtracted gradually on levels ranging from 0% to 100%, with 10% intervals.

This work is shared under a CC BY-SA 4.0 license unless otherwise noted

2.2 Supplementary figures of ADS results

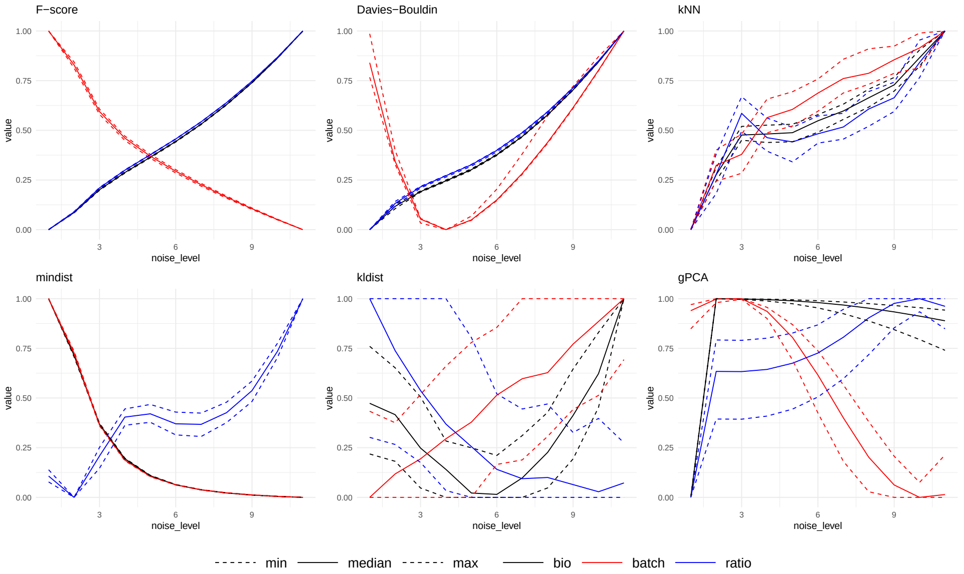

Figure 4: The performance of the evaluation metrics on the levels of the ADS on the biological variation in the multinomial resampled datasets of the in-house colon cancer data. A good metric here shows a monotonous increase in the ratio of biological and batch effects, corresponding to the decrease in the separation between the groups. The decrease in the measured batch effects can be attributed to minor correlation between the biological and batch groups.

This work is shared under a CC BY-SA 4.0 license unless otherwise noted

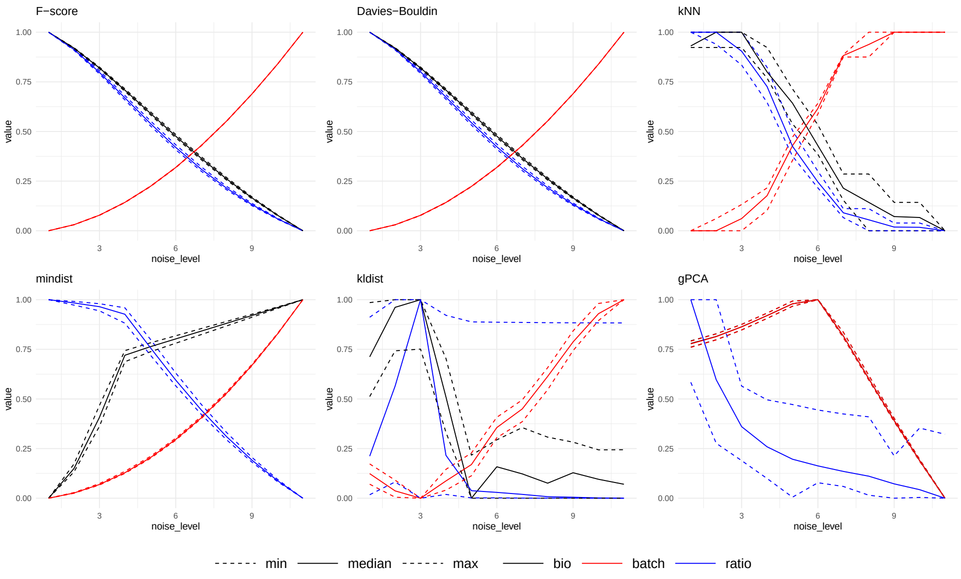

Figure 5: The results of the performance metrics on the multinomial resampled datasets of the HeLa Kyoto cell line data.

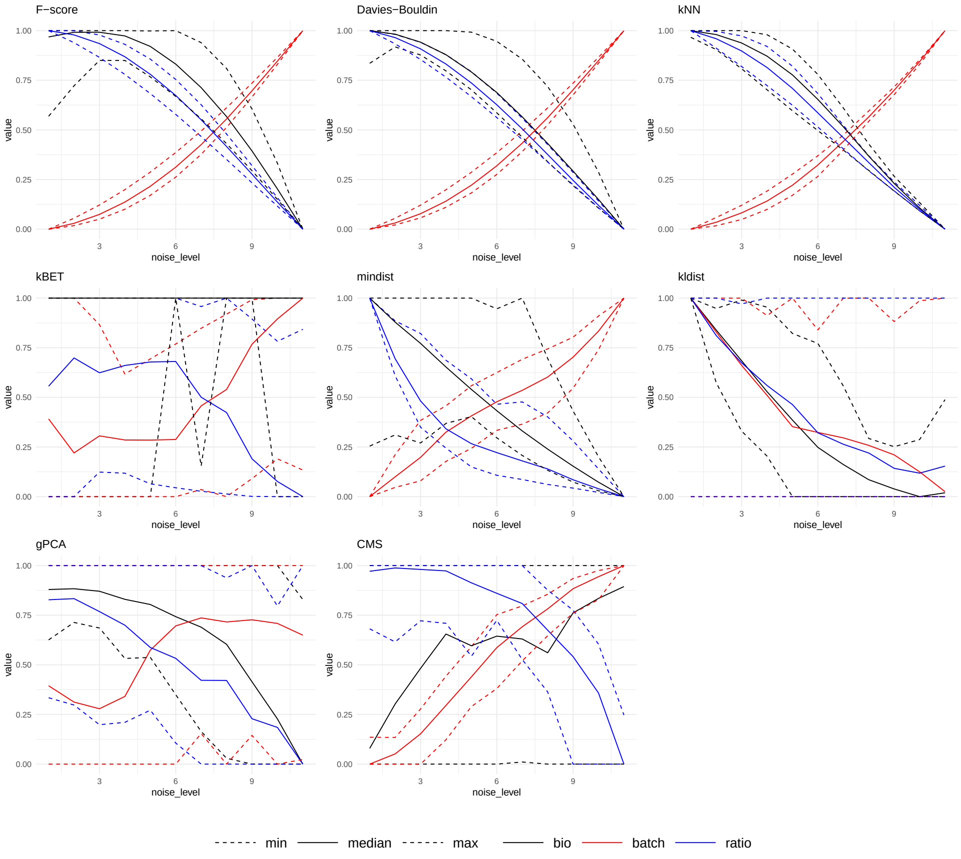

Figure 6: The results of the performance metrics on the multinomial resampled datasets of the mESC single-cell data. The test metric kBET and CMS were included in this comparison, as they were developed specifically for single-cell datasets.

This work is shared under a CC BY-SA 4.0 license unless otherwise noted

3 Supplementary tables

Table 1: The rank correlation score (Spearman's rho) of the biological signal and bio/batch signal ratio for each dataset where the ADS was applied to the biological signal in the data. The results on batch effects are not shown, since they are not relevant in this comparison. Here the rank correlation is negative, hence a lower score is better

Variance model	Data	Signal	Metrics					
			F-score	DB-index	kNN	mindist	kldist	gPCA
Resampling	Inhouse	bio	**-0.996**	**-0.996**	-0.985	**-0.996**	-0.244	0.472
		ratio	**-0.996**	**-0.996**	-0.873	-0.883	0.82	-0.958
	HeLa Kyoto	bio	**-0.996**	**-0.996**	-0.943	0.996	0.461	0.191
		ratio	**-0.996**	**-0.996**	-0.914	-0.638	0.518	-0.845
Bootstrap	Inhouse	bio	**-0.996**	**-0.996**	-0.987	0.996	-0.88	-0.938
		ratio	-0.994	**-0.995**	-0.991	0.419	-0.723	-0.988
	HeLa Kyoto	bio	**-0.996**	**-0.996**	-0.979	0.996	0.044	0.43
		ratio	-0.988	**-0.996**	-0.918	-0.665	0.184	-0.706

Table 2: The mean ranks of each metric across all datasets, for the ADS applied to biological effects. The best ranking metric marked with bolding.

F-score	Davies-Bouldin index	kNN score	mindist	kldist	gPCA
1.75	**1.25**	3.50	5.00	5.75	3.75

This work is shared under a CC BY-SA 4.0 license unless otherwise noted
