# Supplementary figures and images for: Development and comparison of evaluation metrics for batch correction reveals performance differences
Source: Bioinform Adv. 2026 May 21;6(1):vbag142. doi: 10.1093/bioadv/vbag142 (PMC13221981; doi:10.1093/bioadv/vbag142)

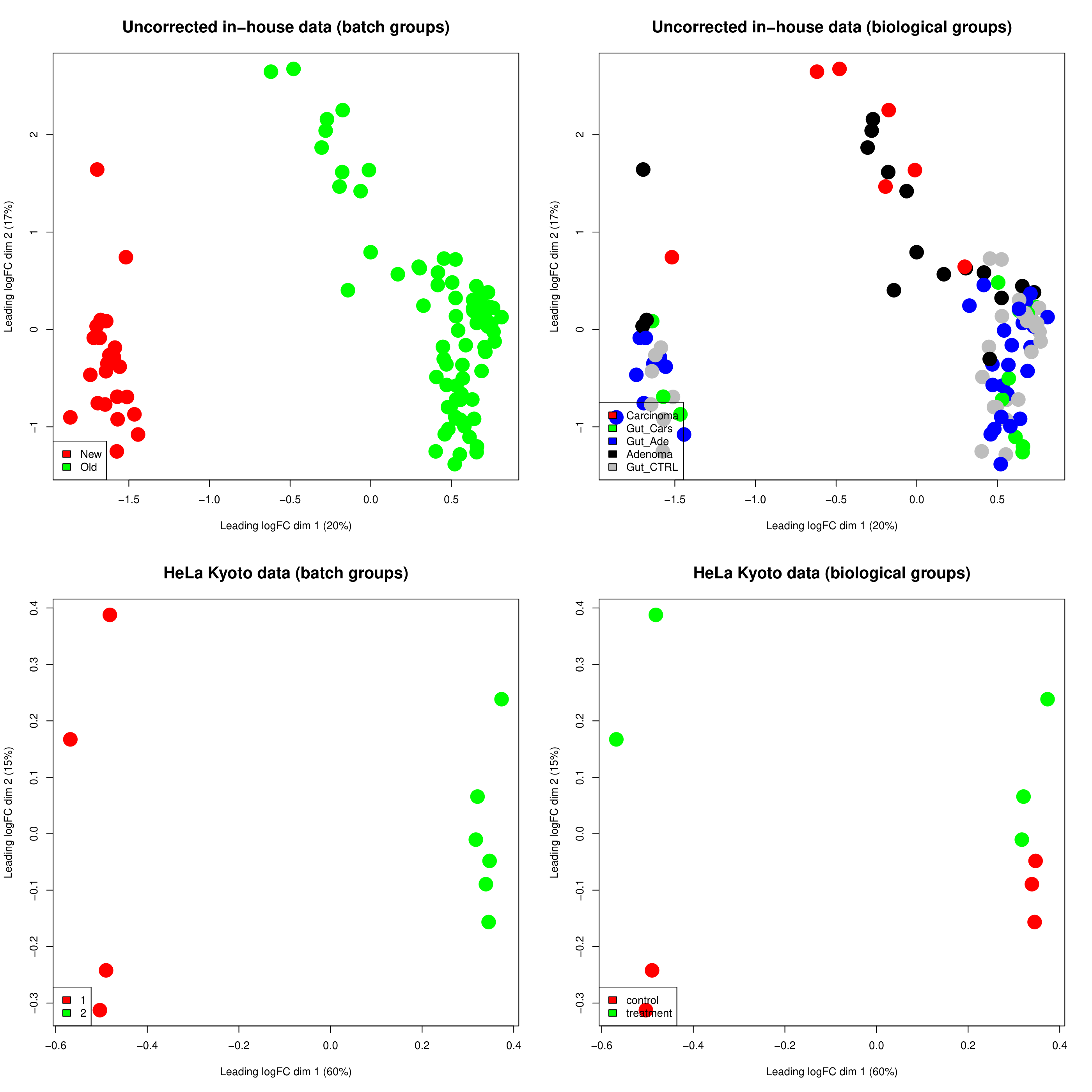

Supplement: vbag142_Supplementary_Data [file vbag142_supplementary_data.zip › Supplementary_figure1.tiff]

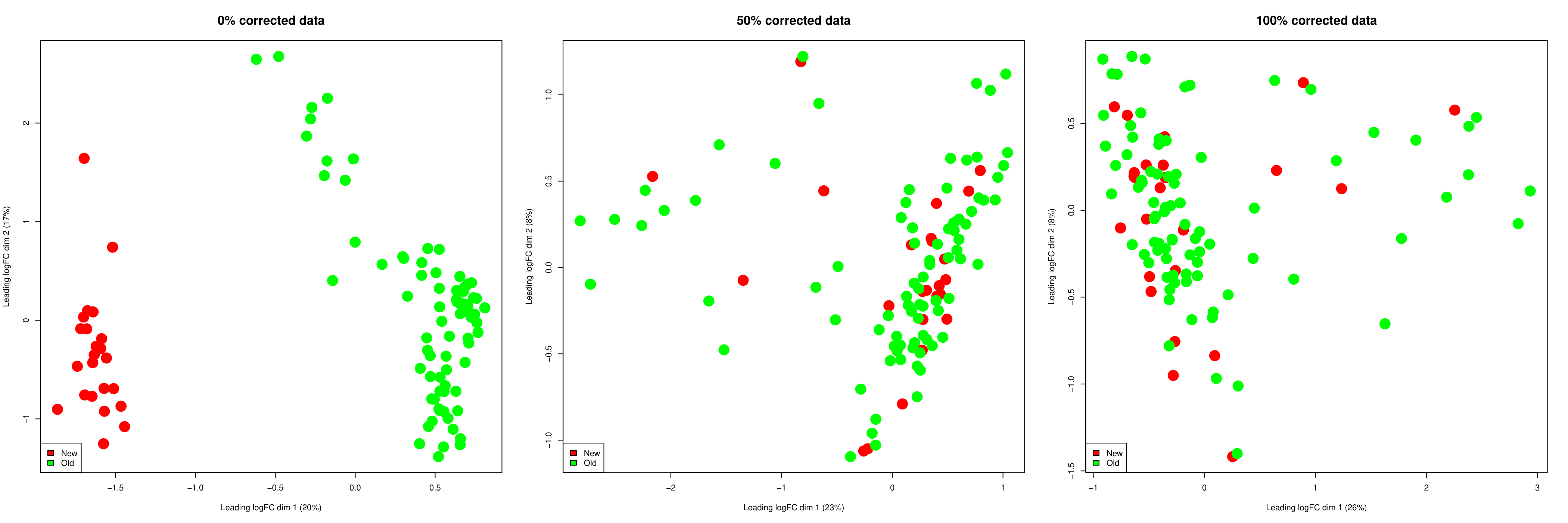

Supplement: vbag142_Supplementary_Data [file vbag142_supplementary_data.zip › Supplementary_figure2.tiff]

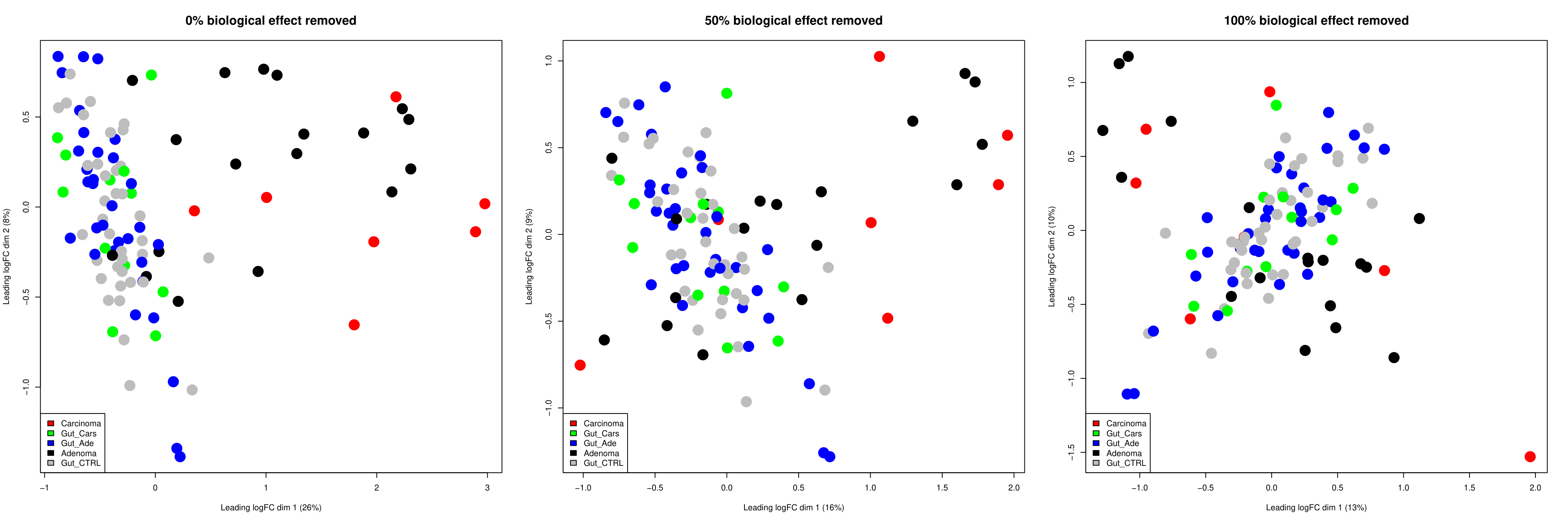

Supplement: vbag142_Supplementary_Data [file vbag142_supplementary_data.zip › Supplementary_figure3.tiff]

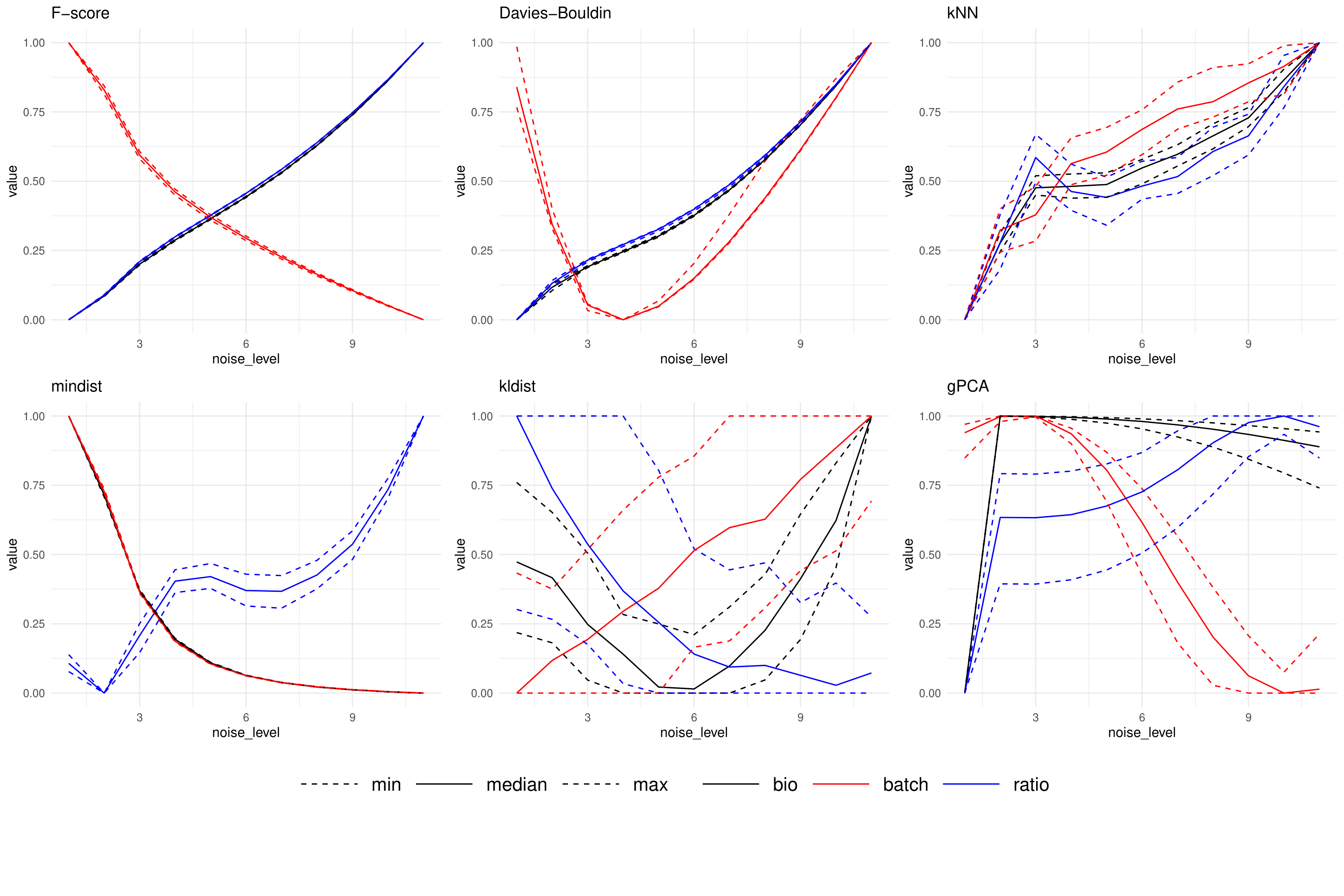

Supplement: vbag142_Supplementary_Data [file vbag142_supplementary_data.zip › Supplementary_figure4.tiff]

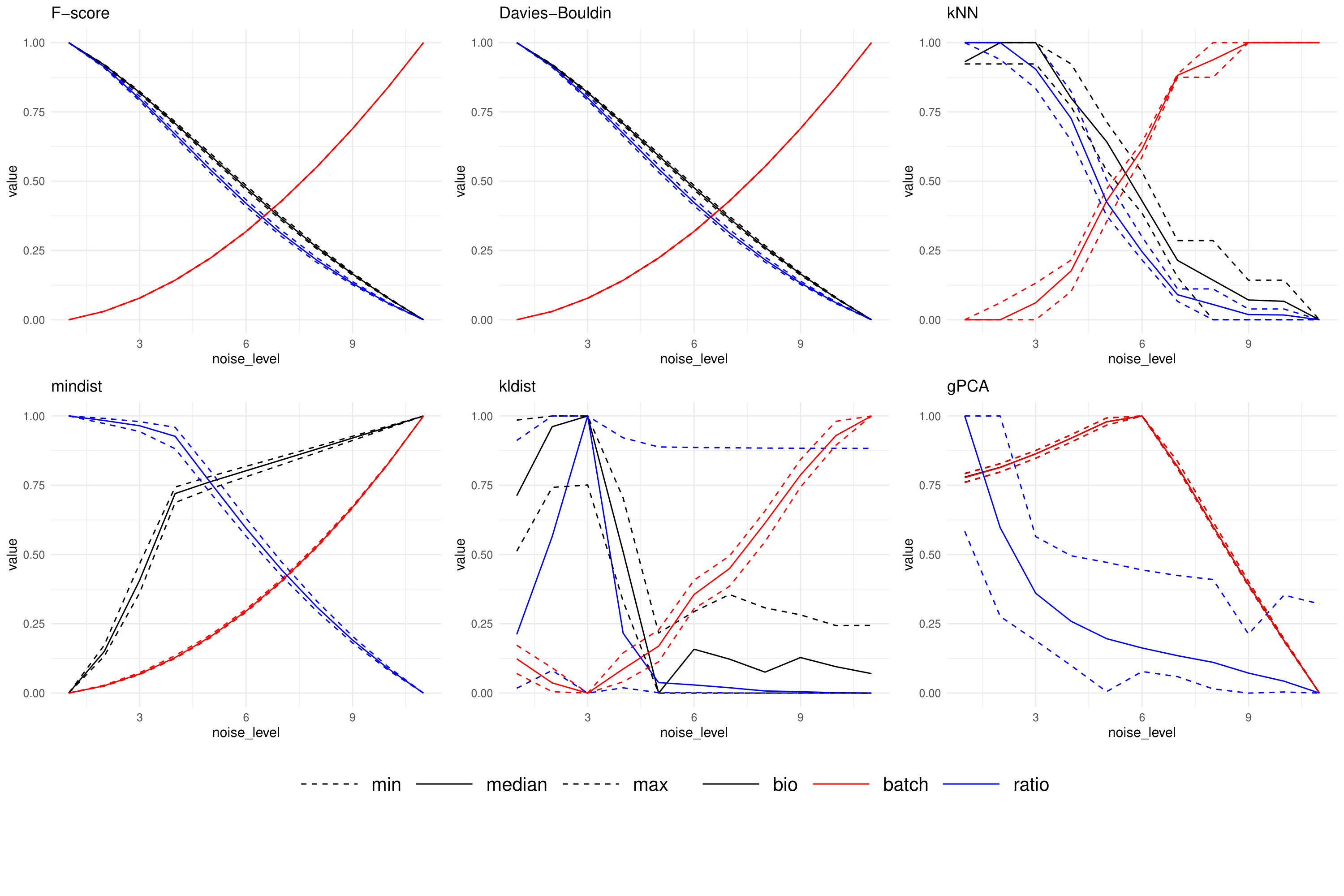

Supplement: vbag142_Supplementary_Data [file vbag142_supplementary_data.zip › Supplementary_figure5.tiff]

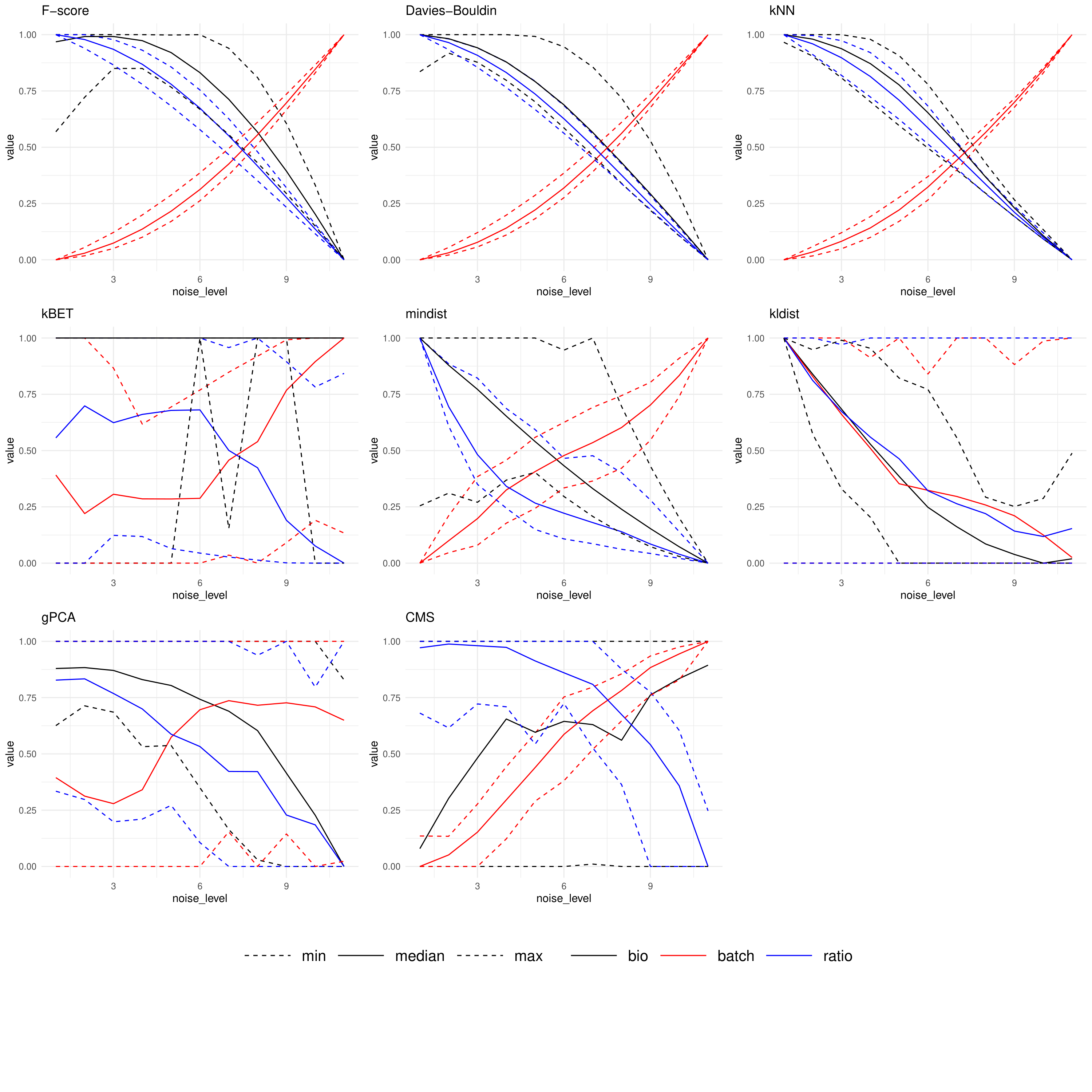

Supplement: vbag142_Supplementary_Data [file vbag142_supplementary_data.zip › Supplementary_figure6[AU].tiff]
